# FLIM of NAD(P)H in Lymphatic Nodes Resolves T-Cell Immune Response to the Tumor

**DOI:** 10.3390/ijms232415829

**Published:** 2022-12-13

**Authors:** Anna V. Izosimova, Marina V. Shirmanova, Vladislav I. Shcheslavskiy, Daria A. Sachkova, Artem M. Mozherov, George V. Sharonov, Elena V. Zagaynova, Diana V. Yuzhakova

**Affiliations:** 1 Institute of Experimental Oncology and Biomedical Technologies, Privolzhsky Research Medical University, 10/1 Minin and Pozharsky Sq., 603005 Nizhny Novgorod, Russia; 2 Institute of Biology and Biomedicine, Lobachevsky State University of Nizhny Novgorod, 23 Gagarin Ave., 603950 Nizhny Novgorod, Russia; 3 Department of Molecular Technologies, Institute of Translational Medicine, Pirogov Russian National Research Medical University, 1 Ostrovityanova, 117997 Moscow, Russia

**Keywords:** FLIM, metabolic coenzyme, NAD(P)H, FAD, autofluorescence, tumor, immune response, T-lymphocyte

## Abstract

Assessment of T-cell response to the tumor is important for diagnosis of the disease and monitoring of therapeutic efficacy. For this, new non-destructive label-free methods are required. Fluorescence lifetime imaging (FLIM) of metabolic coenzymes is a promising innovative technology for the assessment of the functional status of cells. The purpose of this work was to test whether FLIM can resolve metabolic alterations that accompany T-cell reactivation to the tumors. The study was carried out on C57Bl/6 FoxP3-EGFP mice bearing B16F0 melanoma. Autofluorescence of the immune cells in fresh lymphatic nodes (LNs) was investigated. It was found that fluorescence lifetime parameters of nicotinamide adenine dinucleotide (phosphate) NAD(P)H are sensitive to tumor development. Effector T-cells in the LNs displayed higher contribution of free NADH, the form associated with glycolysis, in all tumors and the presence of protein-bound NADPH, associated with biosynthetic processes, in the tumors of large size. Flow cytometry showed that the changes in the NADH fraction of the effector T-cells correlated with their activation, while changes in NADPH correlated with cell proliferation. In conclusion, FLIM of NAD(P)H in fresh lymphoid tissue is a powerful tool for assessing the immune response to tumor development.

## 1. Introduction

Over the past decade, therapies that promote antitumor immune responses have revolutionized the treatment of cancer, resulting in marked and durable responses in subsets of patients across many different tumor types. Despite this, only a subset of patients responds to the therapy, and smaller portions achieve maximum clinical benefit [1]. Investigations of the mechanisms of antitumor immune response are required to enhance the effectiveness of immunotherapy and improve the survival rate of cancer patients.

The functional status of lymphocytes, including their differentiation state and proliferative activity, reflects key changes in the immune system and can be used for diagnostics, prognosis and monitoring of the disease and ongoing therapy, especially, to assess the early response to check-point therapy and assist with quality control on the cells injected into patients for cellular immunotherapies.

Currently, T-cell reactivity is assessed by the expression of surface receptors (such as CD25, CD69, and CD71) or cytokine production (such as IFNγ, transforming growth factor beta, interleukin (IL)-2, IL-4, and IL-17) [2] using flow cytometry, immunohistochemistry, or immunofluorescence, or by transgenic fluorophore expression. However, all these methods are time consuming, require exogenous contrast agents, tissue dissociation and/or fixation. Non-destructive label-free tools are required to fully characterize tumor reactivity of T-cells in their native state. 

Optical imaging using endogenous fluorescence of metabolic cofactors has a great potential as it is non-destructive, provides high spatial and temporal resolution and overcomes the single-use limitation of label-based methods and is independent from the fluorophore concentration.

Cellular energetic and anabolic pathways play a key role in regulating the T-cells’ response to tumors. Metabolic status correlates with their differentiation state, proliferative activity, and a cell subset. Naïve, antigen-activated and memory T-cells have different metabolic profiles [3,4,5]. While the metabolic needs for circulating naïve T-cells are low (low levels of mitochondrial oxidation of glucose-derived pyruvate or fatty acid oxidation (FAO)), antigen-activated T-cells have dramatically increased metabolic demands to support cell growth, proliferation, and effector functions. In a process similar to the aerobic glycolysis (often termed the Warburg Effect) that is observed in cancer cells, activated T-cells exhibit elevated rates of glycolytic activity and increased amino acid metabolism, pentose phosphate pathway (PPP) and fatty acid synthesis. Memory T-cells, like naïve T-cells, are metabolically quiescent cells that use FAO and oxidative phosphorylation (OXPHOS) for energy production. Furthermore, each lineage subset of T-cells has a unique metabolic profile. In the CD4+ compartment, inflammatory effector cells such as Th1, Th2 and Th17 have been found to be glycolytic and reliant on anabolic signaling and metabolic pathways, while regulatory FoxP3 + T-cells have been characterized as utilizing FAO and OXPHOS [3,4,6]. Therefore, the knowledge of the metabolic features in effector and suppressive T-cell subsets offer promising opportunities for selective correction of the immune responses and improve the effectiveness of immunotherapy [7].

It is widely shown that autofluorescence intensities and lifetimes of the metabolic coenzymes such as reduced NAD(P)H and oxidized flavin adenine dinucleotide FAD provide an estimate of cellular metabolic activity (Supplementary Figure S1). These cofactors act as metabolic electron carriers and exist in the reduced (NAD(P)H, FADH2) and oxidized (NAD+/NADP+, FAD) forms in a cell. Out of the redox cofactors, only NAD(P)H and FAD are autofluorescent, with NAD(P)H typically exhibiting higher fluorescence intensity within cells compared to FAD. Perturbations in various energetic and anabolic pathways can result in the change of the autofluorescence parameters of the metabolic coenzymes, which can be observed on a single-cell level using fluorescence lifetime imaging (FLIM) microscopy [8,9,10,11]. 

Fluorescence lifetime imaging has been extensively used for metabolic investigations of cancer and other diseases [12,13,14,15,16,17]; however, the studies of immune cells using metabolic autofluorescence imaging is a very recent trend, and there are only a few studies so far [8,9,10,11,12,13,14,15,16,17,18,19,20]. 

In the present study we have investigated whether autofluorescence parameters of NAD(P)H and FAD assessed from two-photon FLIM microscopy in the effector T-cells are sensitive to their reactivation to the tumor development. 

The study was carried out on C57Bl/6 FoxP3-EGFP mice bearing B16F0 melanoma. Melanoma is known to be one of the main targets of studies on immune-checkpoint inhibitors due to its high immunogenicity [21,22]. Experiments were performed on freshly excised LNs on 10–11th and 14–15th days after tumor implantation. The time points and, respectively, tumor sizes were selected based on our previous studies on B16F0 melanoma model and immune check-point inhibitors [23,24,25]. The LNs from intact mice without tumors served as a control. For the first time, the effector T-cells were analyzed within freshly excised lymphoid tissue to simulate the native conditions with in vivo stimulation of the immune system by tumor development. Activation and proliferation of the effector T-cells was confirmed by flow cytometry.

## 2. Results

### 2.1. Two-Photon Microscopy of NAD(P)H and FAD Fluorescence Intensity 

As only reduced NAD(P)H and oxidized FAD are fluorescent, the ratio of their fluorescence intensities is used to measure the overall redox state of the cells. First, we investigated whether the fluorescence intensity of metabolic coenzymes and the intensity-based FAD/NAD(P)H redox ratio differed between the intact and tumor-bearing mice. 

**Figure 1:**
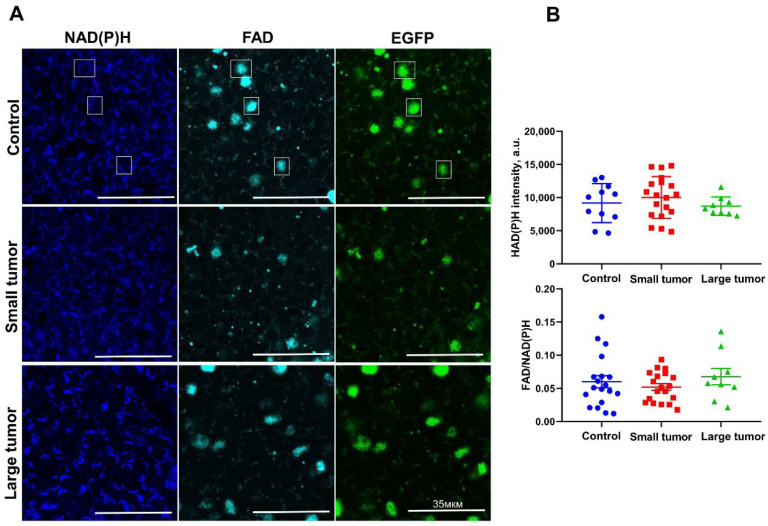
Fluorescence intensity of NAD(P)H and FAD in LNs of intact and tumor-bearing mice. (**A**) Representative intensity-based images in NAD(P)H (blue, em = 450–490), FAD (azure, em = 500–550) and EGFP (green, em = 500–570) channels. Examples of the regulatory FoxP3+ CD4+ T-cells, stably expressing EGFP, are marked by the white squares in all channels. (**B**) Quantification of fluorescence intensity-based redox ratio FAD/NAD(P)H and fluorescence intensity of NAD(P)H. Scatter dot plot displays the measurements for individual animals (dots), the mean and SEM (horizontal lines).

The focus of the present study was on the cell subsets that support the immune response against tumors. Therefore, the regulatory FoxP3 + T-cells that act to suppress the immune response were detected by EGFP fluorescence and excluded from the analysis (Figure 1A). 

No difference in the FAD/NAD(P)H ratio and the NAD(P)H fluorescence intensity was found between mice with and without tumors as well as between mice with small and large tumors (Figure 1B). In all the groups, a high degree of heterogeneity was observed between LNs. 

Therefore, fluorescence intensity of the metabolic cofactors failed to resolve metabolic changes in T-cells’ response to tumor growth.

### 2.2. FLIM of NAD(P)H 

In the next step, autofluorescence lifetime parameters of NAD(P)H were analyzed in the immune cells by FLIM during tumor development. The data processing on the lifetime of FAD failed due to low number of photons (<1000) at binning factor of 3. To obtain a sufficient number of photons (≥5000) to build the decay curve, a binning factor of 10 would be required, which is not desirable to use due to a significant deterioration in spatial resolution.

**Figure 2:**
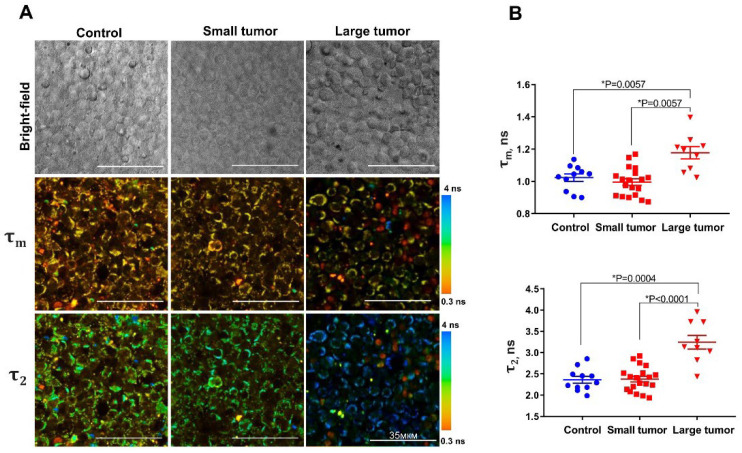
FLIM of NAD(P)H in LNs of intact and tumor-bearing mice. (**A**) Representative FLIM images of the mean fluorescence lifetime τ_m_ and long lifetime component τ_2_. (**B**) Quantification of fluorescence lifetime parameters. Scatter dot plot displays the measurements for individual animals (dots) and the mean and SEM (horizontal lines).

First, we processed the NAD(P)H FLIM data using conventional bi-exponential fitting, where the lifetime τ_1_ and relative amplitude *α*_1_ were attributed to free NADH, and τ_2_ and *α*_2_ were attributed to both protein-bound NADH and NADPH (Figure 2).

In the control group without tumors, the NAD(P)H fluorescence lifetime parameters in T-cells were typical, with τ_1_ ~0.45 ns, τ_2_ ~2.35 ns, *α* _1_ ~69.35%, *α* _2_ ~30.64%, and τ_m_ ~1.0 ns.

In the “Large tumor” group, we demonstrated a statistically significant increase in the mean fluorescence lifetime (τ_m_) compared to the “Control” (*p* = 0.0057) and “Small tumor” (*p* = 0.0057) groups due to elongation of the τ_2_ value.

The most probable reason for elongation of τ_2_ is an increased contribution of phosphorylated form (NADPH), that has a rather long fluorescence lifetime (~4.4 ns) [10]. To obtain the data on NADPH, the “Large tumor” group was processed using a three-exponential decay model. Upon three-exponential fitting, the lifetime τ_2_ and relative amplitude *α*_2_ are attributed to protein-bound NADH, and τ_3_ and *α*_3_ are attributed to the protein-bound NADPH. 

Significant heterogeneity was detected in the distribution of protein-bound NADPH (*α*_3_) fractions among T-cells in individual LNs of mice with large tumors (Supplementary Figure S2A). We reliably registered NADPH only in a part of the cell population (23–83% of cells) in each LN sample, those in which τ_2_ during bi-exponential fitting was in the range 3.1–4.4 ns. In these cells, fluorescence lifetime τ_3_ was 3.65 ± 0.3 ns and the relative amplitude *α*_3_ was 10.1 ± 0.47%. In the rest of the cells, although some elongation of τ_2_ (to 2.7–3.1 ns) was observed, the contribution of NADPH to the overall NAD(P)H pool was insufficient to extract it from the three-exponential fit. As for dispersion of the protein-bound NADH (a_2_) fraction in the individual LNs, it was similar in mice with and without tumors (Supplementary Figure S2B).

**Figure 3:**
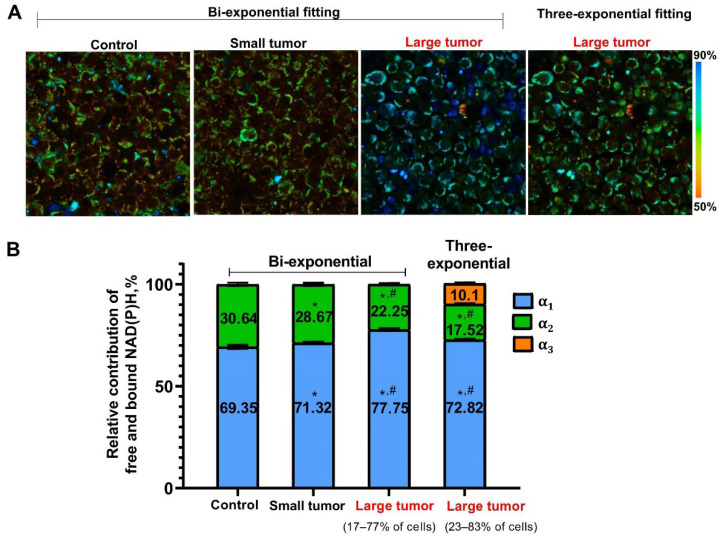
Relative contributions of NAD(P)H in LNs of intact and tumor-bearing mice. (**A**) Representative FLIM images of the relative amplitude of free NADH *α*_1_, upon bi- (all groups) and three- (“Large tumor” group) exponential fitting. (**B**) Quantification of relative contributions of free and bound NADH (*α*_1_, *α*_2_) and NADPH (*α*_3_). The data are presented as mean ± SEM. * *p* < 0.03, compared to “Control” group; ^#^ *p* < 0.0001, compared to the “Small tumor” group. Three-exponential fitting was applied to the part of cells (28–83%) having long lifetime τ_2_ within each LN; other cells were processed with bi-exponential fitting.

Figure 3 demonstrates the relative contributions of free and bound NADH (*α*_1_, *α*_2_) and, if appropriate, NADPH (*α*_3_) in all groups of mice with a high heterogeneity of NADPH-α_3_ distribution in the “Large tumor” group. We detected an increase in relative amplitude of free NADH *α*_1_ and, respectively, decrease in protein-bound NADH *α*_2_ in LNs of all tumor-bearing mice in comparison with control mice, which indicated a shift to a more glycolytic metabolism in reactive T-cells. Concerning the “Large tumor” group, irrespective of the contribution of NADPH, the *α*_1_ and *α*_2_ values changed in both subgroups of T-cells irrespective of the contribution of NADPH.

Therefore, FLIM of the immune cell autofluorescence demonstrated the sensitivity of the NAD(P)H lifetime parameters to the tumor development. The most notable changes were an increase in relative amplitude of free NADH *α*_1_ in LNs of all tumor-bearing mice with more pronounced changes in the case of large tumors and appearance of NADPH contribution *α*_3_ in some T-cells of mice with large tumors.

### 2.3. Expression of Activation and Proliferative Markers by the Effector T-Cells

To verify the immune response to the tumor, we evaluated the activation and proliferation in two main effector T-cell subsets: conventional CD4+ T helper (Th) and CD8+ T-cells isolated from LN.

**Figure 4:**
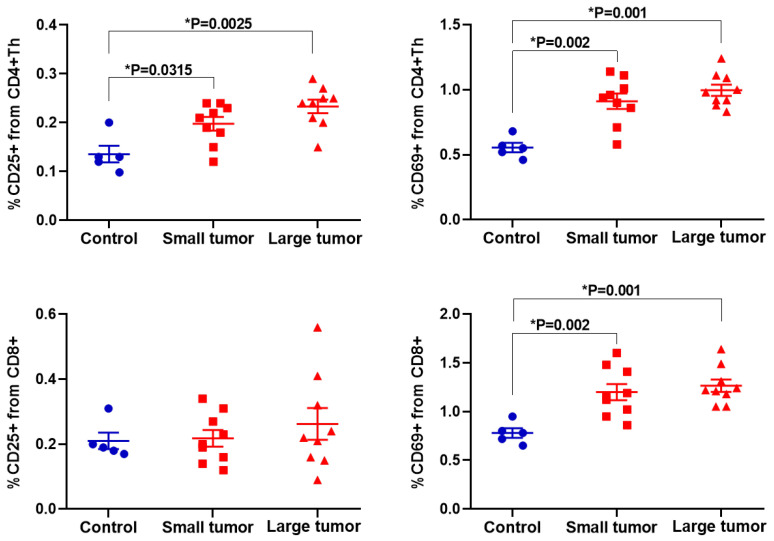
Expression of CD69 and CD25 in live CD4+ Th and CD8+ T-cells. Scatter dot plots display the measurements for individual animals (dots) and the mean and SEM (horizontal lines).

First, we assessed the expression of the cell surface markers of early (CD69) and middle (CD25 (IL-2Rα)) activation in live immune cells (Figure 4). Flow cytometry data demonstrated the pronounced increase in the percentage of CD69+ cells in both CD4+ Th (*p* < 0.002) and CD8+ T-cells (*p* < 0.002) and moderate increase in the percentage of CD25+ among CD4+ Th cells (*p* < 0.03) in all tumor-bearing mice in comparison with “Control” group. “Small tumor” and “Large tumor” groups did not statistically differ from each other.

**Figure 5:**
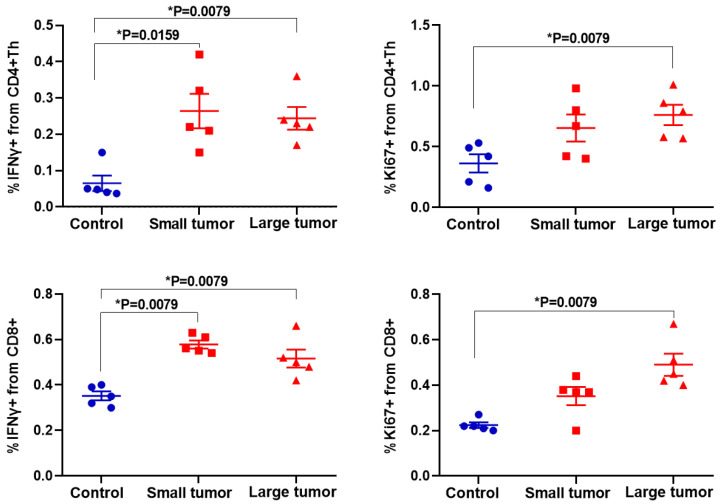
Expression of IFNγ and Ki67 in fixed CD4+ Th and CD8+ T-cells. Scatter dot plots display the measurements for individual animals (dots) and the mean and SEM (horizontal lines).

Furthermore, we evaluated the intracellular expression of activation and proliferative markers IFNγ and Ki67, respectively, by the intracellular staining (ICS) of fixed immune cells (Figure 5). We found a statistically significant increase in production level of IFNγ in both CD4+ Th (*p* < 0.02) and CD8+ T-cells (*p* = 0.0079) in all tumor-bearing mice in comparison with control. 

Concerning the proliferative index Ki67, the statistically significant increase was detected only in the “Large tumor” group (*p* = 0.0079) but not in the “Small tumor” group compared to “Control” in both effector T-cell subsets.

Therefore, the effector T-cells underwent activation in response to tumor development, as expected, and the expression of the main activation markers was slightly more pronounced in the case of tumors of larger size. In addition, tumor development enhanced proliferation of T-cells, which was also higher in the group of large tumors. These results suggest that the observed increase of the free NADH fraction in T-cells, the indicator of glycolytic shift, is likely due to their activation, while the increase of the protein-bound NADPH fraction is associated with their proliferative activity.

## 3. Discussion

One of the main challenges in the field of tumor immunity and cancer immunotherapy is a search for reliable biomarkers and tools for evaluation of the T- and B-cell immune response. Autofluorescence imaging of metabolic coenzymes is an attractive technique because it provides nondestructive label-free examination of dynamic changes of the cell and tissue metabolism both in vitro and in vivo and enables fast data obtaining and processing. Here, we investigated the autofluorescence of the immune cells from fresh LN fragments by the assessment of an intensity-based redox ratio FAD/NAD(P)H and fluorescence lifetime parameters of NAD(P)H by two-photon fluorescence microscopy and FLIM, respectively. It should be noted that the selected time-points for LNs’ excision corresponded to the time that the immune response to the tumor grows up, i.e., before T-cell exhaustion.

Autofluorescence imaging has been extensively used for metabolic investigations of cancer and other diseases. However, its application to probing the functional state of immune cells is a very recent trend. There are only a few works with optical metabolic imaging of the immune cells so far. For example, in 2020–2022 the group by M. Skala reported on in vitro [18] and intravital [26,27] studies of macrophages metabolism in normal and cancerous tissue using the intensity-based redox ratio and FLIM of NAD(P)H and FAD. Moreover, in 2021, they demonstrated the ability of autofluorescence imaging to distinguish between quiescent and activated T-cells, as well as between the subsets of CD4+ and CD8+ cells [19]. Besides, the study by Lemire et al. showed the increased NAD(P)H and FAD fluorescence intensity in stimulated CD4+, CD8+ and B-cells and the possibility to discriminate between the various types of innate and adaptive immune cells using autofluorescence intensity of the coenzymes [20]. However, in both studies the T-cell subsets were extracted from healthy donors and cultured in vitro, and the different activation states were modeled by adding non-antigenic stimuli. 

The feature of our study is that autofluorescence imaging of T-cells was performed within fresh lymphoid tissue, that is under conditions close to natural. Our preliminary results suggested that disaggregation of LNs into the cell suspension changed all the parameters of NAD(P)H fluorescence lifetime (Supplementary Figure S3), which means that the conditions of T-cell maintenance are important. 

Quantification of the optical redox ratio is an old, simple and one of the widely used approaches that is based on the ratio of fluorescence intensity of reduced and oxidized forms of metabolic coenzymes [28]. Using the redox ratio, Walsh et al. demonstrated the difference between quiescent and activated CD3+ T-cells and CD3+ CD8+ cells [19]. Lemire et al. showed the increase in fluorescence intensity of both coenzymes after stimulation of CD4+ and CD8+ T-cells [20]. However, we did not detect any statistically significant differences in the FAD/NAD(P)H ratio and NAD(P)H fluorescence intensity between immune cells from intact and tumor-bearing mice. Inconsistencies between our and literature data can be explained by both different conditions of the experiment (e.g., conditions of T-cell maintenance or different nature of activating stimuli) and limitations of the intensity-based approach. A serious limitation is that fluorescence intensity depends on many factors, apart from concentration of the cofactor, e.g., proximity of cells to the detector, focusing, photobleaching, excitation power, light absorption, and scattering, which significantly complicates the studies on tissues and comparisons between data sets.

It should be noted that the intensity measurements give only general information about the redox status of cells, but do not allow to distinguish different forms of the cofactors (free, protein-bound non-phosphorylated and phosphorylated forms of NADH and “open” and “stacked” conformations of FAD and FNM). Unfortunately, FAD fluorescence was very weak in the LNs, so that FLIM of FAD could not be implemented properly. 

In our study, FLIM allowed sensitive discrimination of NADH and NADPH in T-cells, which provided information about alterations in energetic and anabolic pathways induced by the tumor. Like Walsh et al., we demonstrated a significant increase in free NADH contribution *α*_1_ associated, likely, with glycolysis upregulation in T-cells of tumor-bearing mice compared to intact mice [19]. It is known that T-cells exhibit elevated rate of aerobic glycolysis and glutaminolysis after encounter with an antigen. The T-cell antigen receptor stimulation leads to activation of the phosphatidyl-inositide-3-kinase (PI3K)/Akt/mTORC1 signaling pathway and induction of Myc, which promotes both aerobic glycolysis and increased glutamine metabolism, and drives increased lymphocyte numbers and size [29]. Aerobic glycolysis promotes the production of inflammatory cytokines. The glycolytic intermediate phosphoenolpyruvate has been suggested to promote calcium signaling essential for T-cell activation [30]. Glycolysis is known to maintain acetyl-CoA pools substrates that are necessary for epigenetic promotion of IFNγ gene expression and enhances IFNγ mRNA translation into protein [3,29,31,32].

We assessed the intracellular production of IFNγ by intracellular staining (ICS) and confirmed the significant increase in its expression in both CD4+ Th and CD8+ T-cells in all tumor-bearing mice. Interestingly, *α*_1_ demonstrated significant changes already at an early stage of tumor development (“Small tumor” group), which correlates with flow cytometry data on IFNγ production and the expression of CD25 and CD69. 

The next pool of the reactions includes further conversions of pyruvate and is associated with protein-bound NADH (τ_2_, *α*_2_). Pyruvate can be shuttled into the mitochondria, where it is further broken down by pyruvate dehydrogenase complex (PDC), producing acetyl CoA. Another source of acetyl CoA in mitochondria is FAO. The acetyl CoA molecules can then enter the tricarboxylic acid (TCA) cycle. During all these processes (oxidative decarboxylation of pyruvate, FAO, and TCA) and glutaminolysis reactions (such as conversion of glutamate to α-ketoglutarate and malate to pyruvate), NADH is produced which then donates electrons in Complex I (NADH dehydrogenase) within the electron transport chain (ETC). An alternative fate of pyruvate under anaerobic or aerobic (Warburg effect) conditions is conversion to lactate through lactate dehydrogenase (LDH) with NADH oxidation to NAD+. Consequently, NADH can be bound with a wide range of enzymes including LDH, malate dehydrogenase, citrate synthase, and Complex I of ETC, and its conformational heterogeneity has been shown to be the cause of the high variability in τ_2_ [33,34,35,36].

Last, an alternative way of glucose use is PPP, in which pentose-5-phosphates are produced that are needed for biosynthesis of nucleotides and aromatic amino acids. Furthermore, PPP is a major source of NADPH that is mainly involved in fatty acid synthesis, pyruvate oxidation to malate, and the reduction of glutathione. It is known that activated T-cells as well as other cells with a high proliferative activity, are characterized by enhanced PPP flux [5,6].

Using the three-exponential fitting method, we detected the bound NADPH (τ_3_, *α*_3_) in 23–83% of cells within each LN sample in the “Large tumor” group. Evaluation of the proliferative index Ki67 confirmed an increased proliferative activity of both CD4+ Th and CD8+ T-cells only in “Large tumor” group that correlates with NADPH assay. Previously, NADPH contribution has been observed in several studies using FLIM. For example, it was found in cancer cells undergoing apoptosis [37], human embryonic kidney cells upon metabolic perturbations [10], in stem cells during adipogenic differentiation [38], and in liver tissue [17,39]. In each case, increased NADPH production was associated with different processes. Therefore, this is the first time that NADPH was observed in the immune cells, allowing us to differentiate between T-cells in the early and late stages of tumorigenesis. 

In the rest of the cells (17–77% of the cell within each sample), the increase in τ_2_-NAD(P)H was less pronounced—up to 3.1 ns, which could be due to the low contribution of bound NADPH or binding NADH to different proteins, e.g., to LDH. Sharick et al. recently demonstrated that the lifetime of cellular NADH bound to LDH (~2.4–2.9 ns) is longer than the overall NAD(P)H τ_2_ [36]. 

In conclusion, we observed that tumor progress resulted in significant reprogramming of T-cell metabolism, and the changes increased as tumors grew. The data on FLIM of NAD(P)H, specifically a gradual increase of the free NADH fraction *α*_1_ and appearance of phosphorylated NADPH *α*_3_, reflect the increase of antitumor immune response due to a growing release of tumor antigens. 

## 4. Materials and Methods

### 4.1. Tumor Cell Culturing

A B16F0 mouse melanoma cell line was used in the study. The B16F0 cells were cultured in RPMI-1640 Medium with L-glutamine (Roswell Park Memorial Institute 1640 Medium) with adding of 10% fetal bovine serum (FBS), 1% antibiotic-antimycotic (Gibco, Amarillo, TX, USA) on 25 cm^2^ culture flasks in a CO_2_-incubator at 37 °C, 5% CO_2_ and 85% humidity. Sub-cultivation was performed twice a week by adding 1 mL of trypsin-EDTA (25%) to the plate for 2–5 min. To obtain the tumor model, B16F0 cells were collected for the injection by adding 1 mL of trypsin-EDTA (25%) to the plate for 5 min at 37 °C.

### 4.2. Mouse Tumor Model

The experiments were carried out on 43 transgenic C57Bl/6-FoxP3-EGFP mice (kindly provided by Alexander Rudensky, Sloan Kettering Institute, New York, NY, USA). Transgenic mice were generated on the C57Bl/6 genetic background, by knocking in the chimeric construct of EGFP subcloned into the first exon of FoxP3 gene [40], to identify the regulatory FoxP3+ CD4+ T-cells. 

Tumors were generated by subcutaneous injection of 1 × 10^5^ B16F0 cells suspended in 150 μL phosphate-buffered saline (PBS) into the mouse flank near the inguinal LN. 

For monitoring the changes in autofluorescence parameters in immune cells during tumor development, mice were divided into three groups: “Control” (intact mice without tumor), “Small tumor” (tumor volume from 40 to 250 mm^3^, FLIM on 10–11th days of tumor growth) and “Large tumor” (tumor volume from 250 to 430 mm^3^, FLIM on 14–15th days).

Mice were euthanized by 90% isoflurane on the 10–11th or 14–15th days after tumor implantation. Inguinal LNs were excised immediately after sacrifice under stereomicroscope Leica M50 (Leica, Germany). Mice were immobilized at a prone position on a styrofoam stage with needles and 70% ethanol was used to wet the abdomen and groin area. A median incision was made with surgical scissors in the abdominal cavity from the pubis to the xiphoid process. Using microsurgical scissors, the inguinal LN was excised and divided into two fragments, for FLIM-microscopy and for flow cytometry, respectively.

### 4.3. Two-Photon Fluorescence and FLIM Microscopy

For FLIM, a fragment of LN (~1/3 part of LN, ~1 mm thick) was cut off using microsurgical scissors and placed cut-side down on the glass bottom FluoroDish™ (WPI, Shanghai, China). Otherwise, the LN capsule blocks the fluorescent signal from the immune cells. The LN fragment was covered by a napkin soaked in 0.9% saline to avoid drying and moving of the sample during microscopic imaging. The time from the LN excision to the end of the experiment was no more than 20 min.

Two-photon FLIM was performed on a LSM 880 (Carl Zeiss, Oberkochen, Germany) fluorescence confocal laser-scanning microscope equipped with an FLIM module Simple Tau 152 TCSPC (Becker and Hickl GmbH, Berlin, Germany). The Objective C Plan-Apochromat 40×/1.3 NA Oil DIC M27 was used for image acquisition. Two-photon fluorescence of NAD(P)H and FAD was excited at the wavelengths 750 nm and 900 nm, respectively, and the emissions were detected in the ranges 450–490 nm and 500–550 nm, respectively. Single-photon fluorescence of EGFP was excited at the wavelengths 488 nm and the emission was detected in the range 500–570 nm. 

The intensity-based redox ratio FAD/NAD(P)H was calculated from the corresponding two-photon fluorescence images after subtracting the background.

The FLIM image acquisition time was 60 s. A binning factor of 3 was applied for NAD(P)H to adjust the number of photons to ≥5000 per decay curve. The fluorescence decay curves were fitted with a two- or three-exponential decay model. The goodness of the fit, the χ^2^ value, was from 0.8 to 1. NAD(P)H was analyzed in the cytoplasm of the individual cells by using the ROI option. 

The specific model for the fitting (two or three exponents) was selected based on the obtained values of τ_2_ and τ_3_ upon three-exponential fitting. Whenever they were different from each other by less than the time response of the system (~120 ps), the bi-exponential model was selected.

### 4.4. Flow Cytometry

A fragment of LN was mechanically disaggregated in 100 μm of Miltenyi buffer. For labeling of live cells, the antibodies to the CD3, CD8, CD4, CD25, and CD69 (BD Biosciences, San Jose, CA, USA) were used. The ICS cells were fixed, permeabilized and stained with antibodies CD3, CD4, CD8 (BD Biosciences) and IFNγ (Miltenyi Biotec, Bergisch Gladbach, Germany) according to the recommendations of the manufacturer (Inside Stain Kit, Miltenyi Biotec). Labeled cells were analyzed on a BD FACSAria III cell sorter (BD Biosciences, San Jose, CA, USA). Data were analyzed using FlowJo software (BD/Treestar, Ashland, OR, USA). 

### 4.5. Statistical Analysis

The mean values (M) and standard error of the mean (SEM) were calculated. Differences between groups were analyzed using the Mann–Whitney U test in GraphPad Prism 7.00 (GraphPad, San Diego, CA, USA) software. A *p* value ≤ 0.05 was considered statistically significant.

## 5. Conclusions

We observed that tumor progress resulted in significant reprogramming of T-cell metabolism. The data on FLIM of NAD(P)H in effector T-cells displayed a gradual increase of the free NADH fraction α_1_, the form associated with glycolysis, and the appearance of protein-bound NADPH, associated with biosynthetic processes, in the tumors of large size. Flow cytometry showed that the changes in the free NADH fraction of the effector T-cells correlate with their activation, while bound NADPH correlated with cell proliferation. Therefore, we have demonstrated that FLIM of NAD(P)H in fresh lymphoid tissue can assess the immune response to tumor development. Our future work will be focused on validation of the metabolic FLIM of immune cells as a tool to evaluate the efficacy of immunotherapy.

## Supplementary Materials

The supporting information can be downloaded at: https://www.mdpi.com/article/10.3390/ijms232415829/s1.
