# Supplementary material for: FLIM of NAD(P)H in Lymphatic Nodes Resolves T-Cell Immune Response to the Tumor
Source: Int J Mol Sci. 2022 Dec 13;23(24):15829. doi: 10.3390/ijms232415829 (PMC9779489; doi:10.3390/ijms232415829)
Supplement: Supplementary file 1 [file ijms-23-15829-s001.zip › ijms-2089333-supplementary.pdf]

Supplementary Material

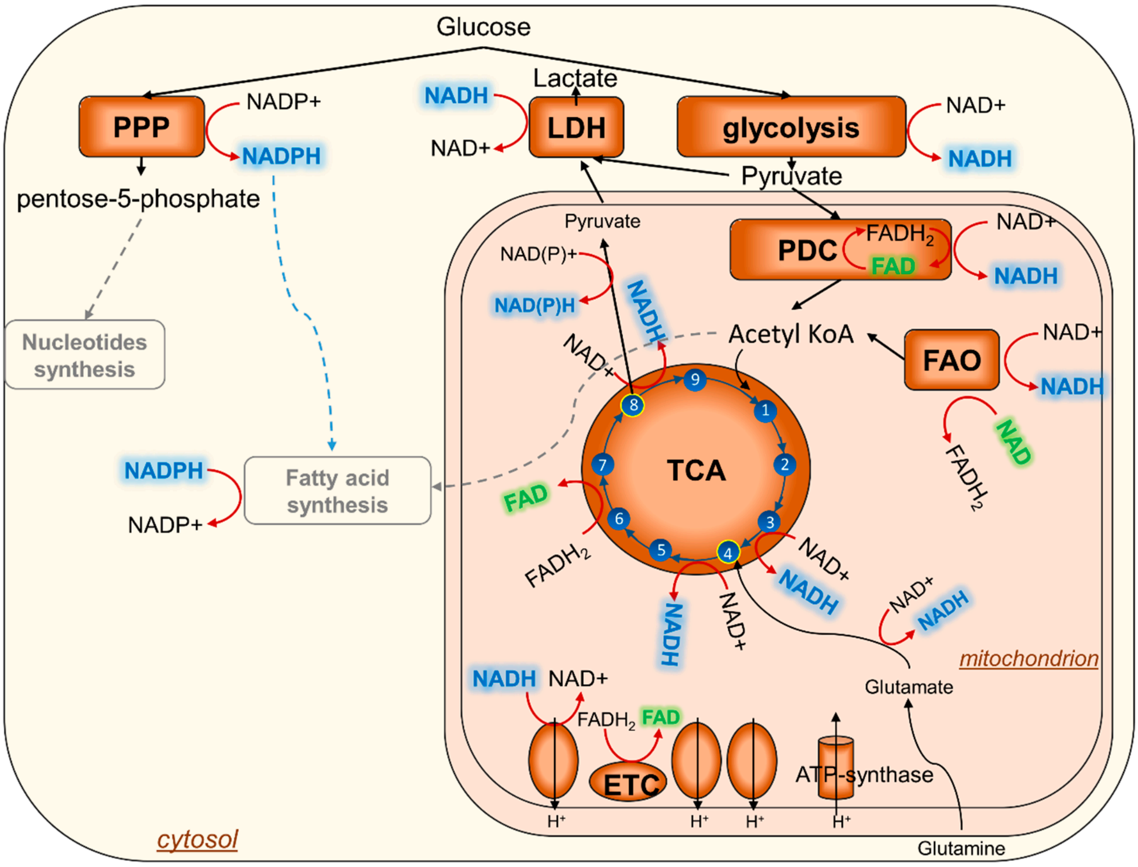

Figure S1: Simplified schematic of the roles NAD(P)H and FAD play in cell metabolism. Blue and green colors indicate the fluorescent forms of coenzymes NAD(P)H and FAD respectively. In TCA 1-9 indicates correspondingly citrate, cis-aconitate, D-isocitrate, α-ketoglutarate (participates in glutaminolysis), succinyl-CoA, succinate, fumarate, malate (participates in glutaminolysis), oxaloacetate.

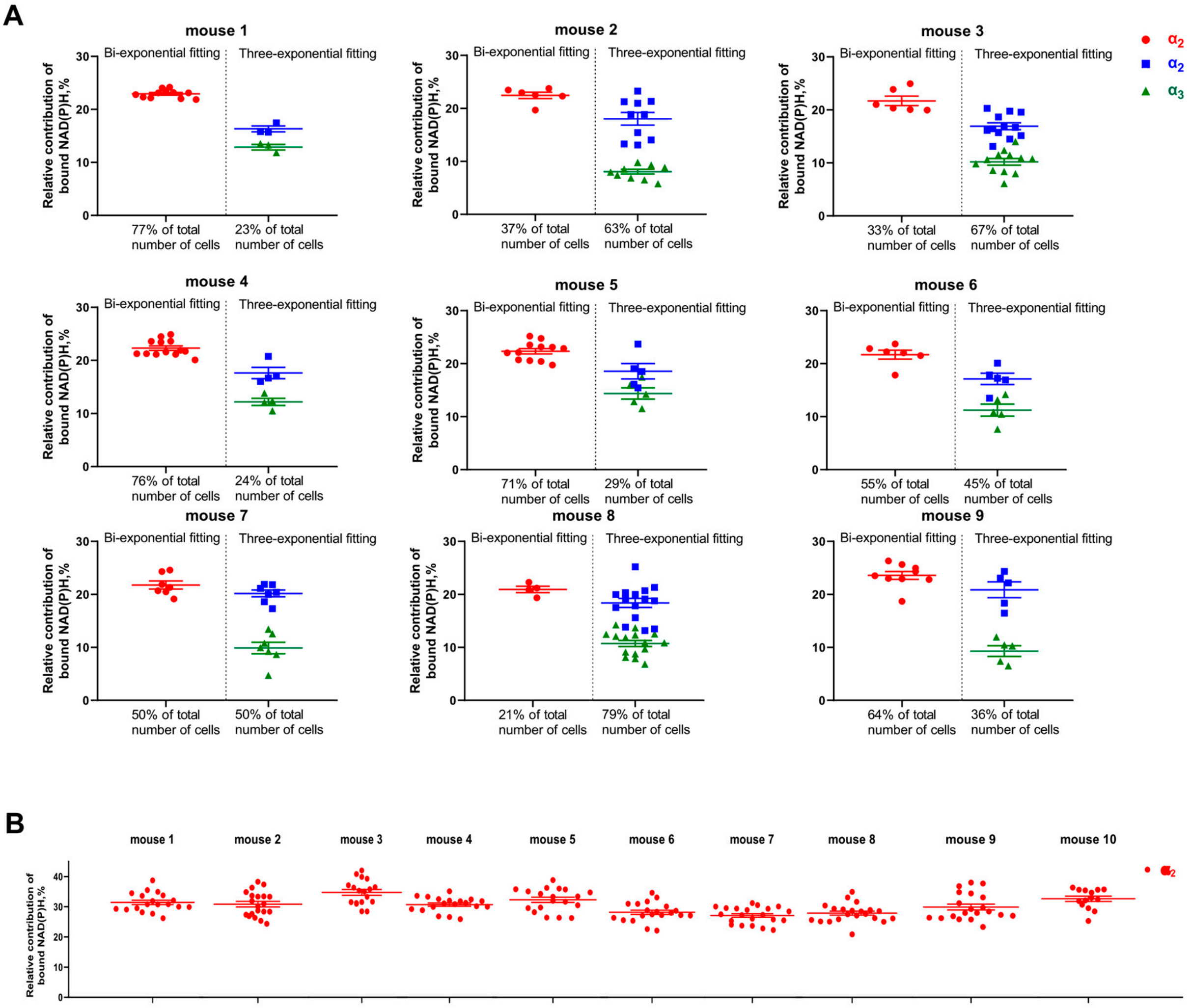

Figure S2: Quantification of the relative contributions of bound NADH (α2) and, if appropriate, NADPH (α3) upon bi- or three-exponential fitting within each LNs in "Large tumor"(A) and control (B) group. Scatter dot plot displays the measurements for individual cells (dots) and the mean and SEM (horizontal lines).

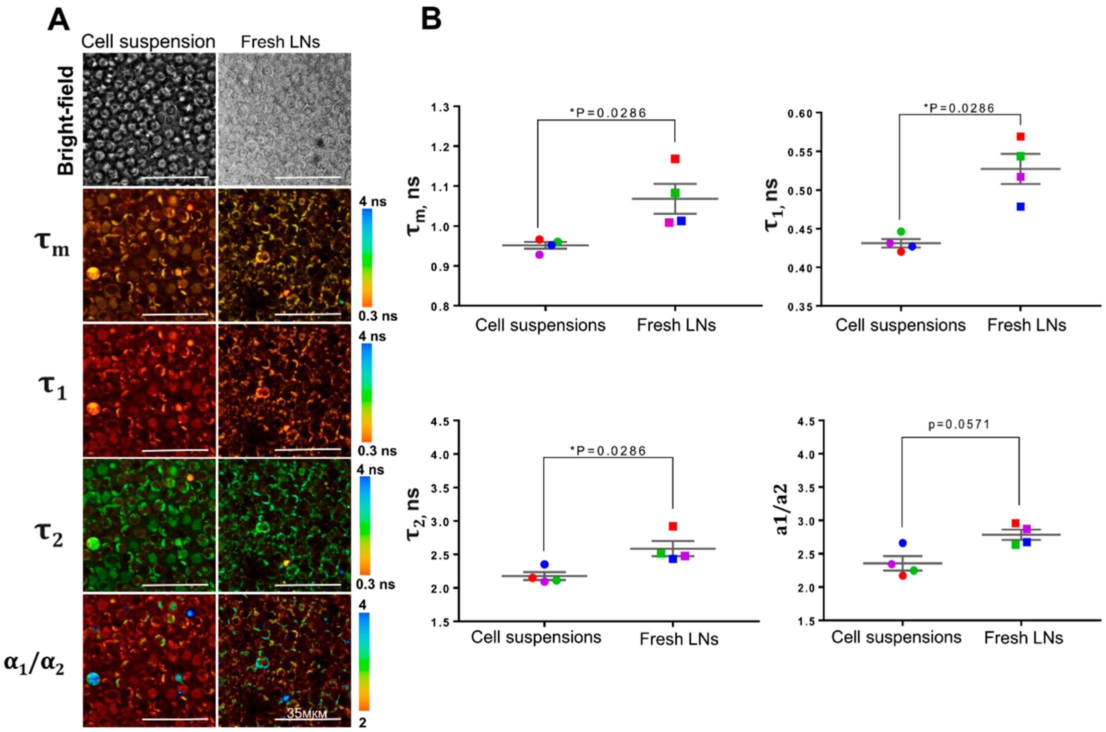

Figure S3: FLIM of NAD(P)H in immune cell suspensions and fresh LNs of intact and tumor-bearing mice. (A) Representative FLIM images of NAD(P)H fluorescence lifetime parameters. (B) Quantification of fluorescence lifetime parameters. Scatter dot plot displays the measurements for individual animals (dots) and the mean and SEM (horizontal lines). The same mice are marked with the same color in the suspension and LN groups.
